# Searching for Multi-Targeting Neurotherapeutics against Alzheimer’s: Discovery of Potent AChE-MAO B Inhibitors through the Decoration of the 2*H*-Chromen-2-one Structural Motif

**DOI:** 10.3390/molecules21030362

**Published:** 2016-03-17

**Authors:** Leonardo Pisani, Roberta Farina, Ramon Soto-Otero, Nunzio Denora, Giuseppe Felice Mangiatordi, Orazio Nicolotti, Estefania Mendez-Alvarez, Cosimo Damiano Altomare, Marco Catto, Angelo Carotti

**Affiliations:** 1 Dipartimento di Farmacia—Scienze del Farmaco, Università degli Studi di Bari “Aldo Moro”, via E. Orabona, 4, I-70125 Bari, Italy; leonardo.pisani@uniba.it (L.P.); roberta.farina@uniba.it (R.F.); nunzio.denora@uniba.it (N.D.); giuseppe.mangiatordi@uniba.it (G.F.M.); orazio.nicolotti@uniba.it (O.N.); cosimodamiano.altomare@uniba.it (C.D.A.); 2 Grupo de Neuroquimica, Departamento de Bioquimica y Biologia Molecular, Facultad de Medicina, Universidad de Santiago de Compostela, San Francisco I, E-15782 Santiago de Compostela, Spain; ramon.soto@usc.es (R.S.-O.); estefania.mendez@usc.es (E.M.-A.)

**Keywords:** Alzheimer’s disease, cholinesterase inhibitors, coumarins, MAO inhibitors, multi-target-directed ligands

## Abstract

The need for developing real disease-modifying drugs against neurodegenerative syndromes, particularly Alzheimer’s disease (AD), shifted research towards reliable drug discovery strategies to unveil clinical candidates with higher therapeutic efficacy than single-targeting drugs. By following the multi-target approach, we designed and synthesized a novel class of dual acetylcholinesterase (AChE)-monoamine oxidase B (MAO-B) inhibitors through the decoration of the 2*H*-chromen-2-one skeleton. Compounds bearing a propargylamine moiety at position 3 displayed the highest *in vitro* inhibitory activities against MAO-B. Within this series, derivative **3h** emerged as the most interesting hit compound, being a moderate AChE inhibitor (IC_50_ = 8.99 µM) and a potent and selective MAO-B inhibitor (IC_50_ = 2.8 nM). Preliminary studies in human neuroblastoma SH-SY5Y cell lines demonstrated its low cytotoxicity and disclosed a promising neuroprotective effect at low doses (0.1 µM) under oxidative stress conditions promoted by two mitochondrial toxins (oligomycin-A and rotenone). In a Madin-Darby canine kidney (MDCK)II-MDR1 cell-based transport study, Compound **3h** was able to permeate the BBB-mimicking monolayer and did not result in a glycoprotein-p (P-gp) substrate, showing an efflux ratio = 0.96, close to that of diazepam.

## 1. Introduction

Nowadays, a growing share of the elderly population experiences the heavy burden of cognitive decline associated with severe neurodegenerative disorders (NDs). In this context, a central role is occupied by Alzheimer’s disease (AD), being the first cause of age-related dementia with a worrisome projection of around 100 million patients worldwide by 2050. In people suffering from AD, learning deficits along with the impairment of memory and language skills result from the disruption of cholinergic transmission in hippocampal areas [1]. As a consequence of the so-called “cholinergic hypothesis”, up to date, the pharmacological therapy of AD has been essentially based on the restoration of adequate levels of acetylcholine (ACh). Thus, the majority of registered drugs for the curing of AD belongs to the class of acetylcholinesterase (AChE) inhibitors (*i.e.*, rivastigmine, galantamine and donepezil) that are able to counteract ACh depletion by reducing its degradation catalyzed by AChE [2]. Apart from AChE blockade, a drug with a different mechanism of action (memantine), which has antagonism to NMDA receptors, has more recently been introduced for the curing of moderate to severe forms of AD [3]. However, none of these medicines is able to slow down or reverse the neuronal degeneration, and all of them exert only symptomatic relief. Despite huge efforts performed by researchers, the curing of AD still represents a challenging task, and a real disease-modifying therapy is still needed. As for many NDs, converging and rising evidences shed light on the multifaceted etiology of AD [4]. This means that simultaneous aberrations, including metal unbalance, protein misfolding/aggregation and production of toxic radicals among others, trigger and/or sustain the neurotoxic cascade ultimately leading to neuronal death. By means of modulating two or more targets involved in the onset and/or progression of the disease, the multi-target strategy has been regarded as an innovative tool for discovering neurotherapeutics against AD [5]. In recent years, programs hitting both production and aggregation of β-amyloid and hyperphosphorylated tau protein failed to identify agents with therapeutic efficacy [6]. Thus, the attention has been shifted to other relevant pathophysiological mechanisms. In particular, oxidative stress has been considered a downstream event anticipating the deposition of amyloid plaques and neurofibrillary tangles [7]. Deregulation of endogenous detoxification redox systems and over-production of radical species lead to lipid peroxidation and nucleic acid mutations. In this context, a neuroprotective activity against oxidative stress has been claimed for new chemical entities able to inhibit monoamine oxidases (MAOs) [8]. These FAD-dependent enzymes catalyze the oxidative deamination of amines deriving from diet (e.g., tyramine), physiological neuronal pathways (e.g., several neurotransmitters, such as catecholamines) or drug assumption. The catalytic cycle produces hydrogen peroxide, a harmful by-product capable of triggering reactive oxygen species (ROS) release. Two isoforms (MAO-A and -B) differing in sensitivity to substrate and inhibitors are known [9]. In particular, the MAO-B isoform has received great attention. In fact, it predominates in CNS, and many studies reported its increased activity in brains affected by AD [10]. Thus, the simultaneous inhibition of MAO-B, as well as AChE enzymatic activity represents an innovative therapeutic weapon [8] and multifunctional molecules able to reduce radicals’ formation and to tackle ACh decrease may be beneficial to hamper the neurotoxic cascade in AD.

Coumarin (2*H*-chromen-2-one) is a nature-friendly heterocycle, easy to be functionalized with a high degree of potential chemical diversity [11]. As we recently reported, its planar backbone can be efficiently lodged into the MAO-B catalytic site [12,13] and is able to interact with the peripheral anionic site (PAS) of AChE [14]. Aiming at identifying novel multipotent compounds with dual AChE-MAO-B inhibitory activity [15], herein, we investigated the chemical decoration of the 2*H*-chromen-2-one core around the less explored position 3, by introducing variously-substituted protonatable basic moieties. On the other side, a lower variability was explored at positions 6 and 7 by studying the effect of methoxy groups [16]. *In vitro* enzymatic screening towards ChEs and MAOs led us to identify a dual inhibitor (derivative **3h**) endowed with a potent and selective MAO-B inhibitory potency in the nanomolar range (IC_50_ = 2.8 nM) together with a moderate AChE affinity (IC_50_ = 8.99 µM). Hit Compound **3h** was submitted to transport studies in Madin-Darby canine kidney (MDCK)-II permeability model in order to evaluate its ability to cross BBB and penetrate into CNS. Furthermore, cell-based assays were used to assess the neuroprotective effect against different oxidative insults (hydrogen peroxide, oligomycin-A and rotenone) in human neuroblastoma SH-SY5Y cell lines. Therefore, coumarin-based **3h** emerged as a promising neuroprotective agent with a multi-target profile, deserving a deeper investigation as a potential anti-AD therapeutic agent.

## 2. Results and Discussion

### 2.1. Chemistry

**Scheme 1:**
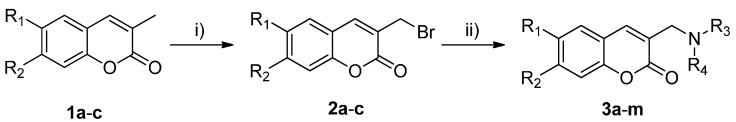
Synthesis of coumarins **3a**–**m**. *Reagents and conditions*: (i) *N*-bromosuccinimide, benzoyl peroxide, CCl_4_, reflux, 2–7 h; (ii) suitable amine, triethylamine (for **3a**,**b**, **3d**–**e**, **3g**, **3l**–**m**) or K_2_CO_3_ (for **3c**, **3f**, **3h**–**k**), DMF at 70 °C (for **3a**, **3l**–**m**) or THF at room temperature (**3b**–**k**), 3.5–24 h.

**Scheme 2:**
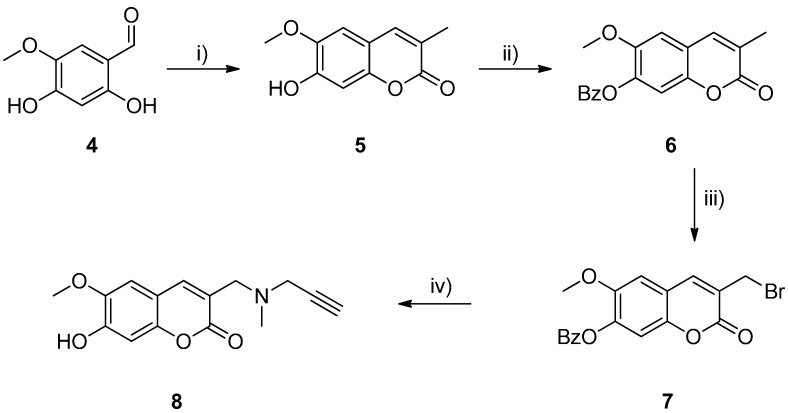
Synthesis of coumarin **8**. *Reagents and conditions*: (i) (carbethoxyethylidene)triphenylphosphorane, *o*-xylene, reflux, 12 h; (ii) triethylamine, benzoyl chloride, dry THF, 5 h; (iii) *N*-bromosuccinimide, benzoyl peroxide, CCl_4_, reflux, 12 h; (iv) *N*-methylpropargylamine, K_2_CO_3_, dry THF, overnight.

The synthetic pathway to final multi-target coumarins **3a**–**m** and **8** is depicted in Scheme 1 and Scheme 2. 3-Methyl-2*H*-chromen-2-ones **1b**,**c** were obtained through a one-pot procedure starting with a Wittig-condensation followed by intramolecular cyclization in refluxing *N*,*N*-diethylaniline [17]. Bromination of **1b**,**c** promoted by *N*-bromosuccinimide (NBS) and benzoyl peroxide (DBP) as the radical initiator afforded intermediates **2a**–**c** that were coupled with the suitable amine to yield Compounds **3a**–**m** (Scheme 1). As depicted in Scheme 2, AlCl_3_-mediated regioselective bis-demethylation of 2,4,5-trimethoxybenzaldehyde furnished aldehyde **4** [18], which was heated at reflux with the appropriate phosphorus ylide in *o*-xylene, thus obtaining coumarin derivative **5**. After protecting the phenolic group, the resulting benzoate ester **6** underwent a radical bromination with NBS and DBP. Final coupling of **7** with *N*-methylpropargylamine in the presence of an excess of potassium carbonate allowed the removal of the benzoate group and the preparation of **8**.

### 2.2. Biological Evaluation

Limited structural modifications were investigated at positions 6 and 7 of the coumarin backbone with the introduction of methoxy groups. On the other side, a higher diversity was explored in the flexible basic moiety where hydrophilic hydrogen-bonding, propargyl and bulkier benzylic groups were inserted. Since cation-π interactions stabilize the complexes of AChE with natural substrate (ACh) and positively-charged inhibitors (e.g., quaternary ammonium salts) at the level of both binding sites (PAS and CAS), a methylene spacer linked the amino group to the heterocyclic scaffold in order to obtain a protonatable base. A ligand-based approach inspired the introduction of propargylamines, a typical structural feature of potent and irreversible MAO-B inhibitors rasagiline [19] and selegiline.

2*H*-Chromen-2-ones **3a**–**m** and **8** were evaluated as inhibitors of electric eel AChE (*ee*AChE), equine serum butyrylcholinesterase (*es*BChE) and rat MAOs (*r*MAO-A and *r*MAO-B). Data are illustrated in Table 1. *In vitro* enzymatic assays on ChEs were accomplished through the well-known spectrophotometric Ellman’s method [20]. MAO inhibitory activities were measured on rat brain mitochondrial homogenates [21]. Propargylamine-bearing inhibitors **3c**, **3f**, **3h** and **8** were pre-incubated with the enzyme preparation for 30 min before the addition of kynuramine and determination of MAO activity. Results are reported in Table 1 as IC_50_ (µM) or as the percentage of inhibition at 10 µM.

Concerning ChEs’ activities, all tested compounds showed no activity or very low inhibitory potency towards BChE, and **3j** was the most active inhibitor (27% inhibition at 10 µM). For the entire series, the affinity towards AChE was moderate, and IC_50_ values were in the range 2.78–14.4 µM, clearly indicating that the reported structural modifications both on the coumarin skeleton and on the basic moiety exerted a limited influence on the activity. Within both small series of unsubstituted and 7-monomethoxy compounds (**3a**–**c** and **3d**–**f**, respectively), the more hindered and lipophilic benzylic group was tolerated by AChE better than the propargyl and hydrophilic amide groups (**3b** > **3a** and **3c**, **3d**, **3e** > **3f**). The presence of the hydrogen bonding benzylamine group yielded the most potent AChE inhibitor (**3j**, IC_50_ = 2.78 µM) belonging to the class of 6,7-dimethoxy derivatives. Regarding MAOs affinity, with the exception of propargylamines **3c**, **3f** and **3h**, the inhibitory activities displayed by compounds herein reported were surprisingly very low, irrespective of the electronic and lipophilic features of the basic chain and coumarin substituents. An interesting effect on activity and isoform selectivity can be observed for methoxy substituents at positions 6 and 7, with MAO-B affinity rising from unsubstituted to mono- and di-methoxy compounds (**3c** < **3f** < **3h**). Interestingly, an opposite trend was performed towards MAO-A (**3h** < **3f** < **3c**), where the affinity drop determined the outstanding selectivity displayed by **3h**. The dramatic loss of affinity for 6-methoxy-7-hydroxycoumarin **8** might be ascribed to the possibility of forming an intramolecular hydrogen bond responsible for the lack of polar interactions, which negatively influenced the binding of **8** with both MAO isoforms.

**Figure 1:**
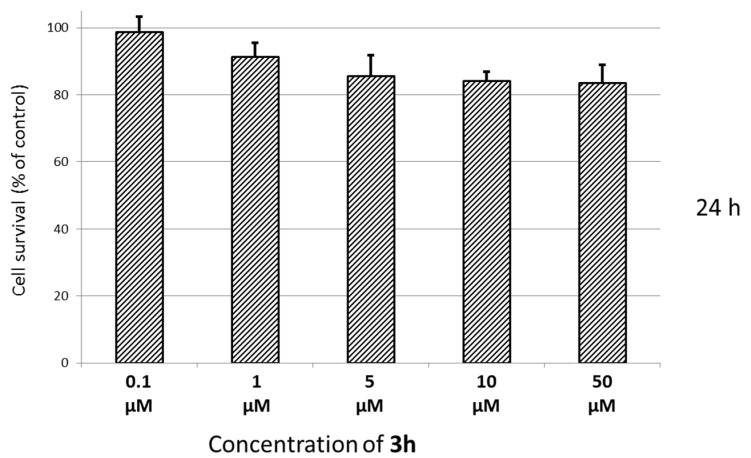
Viability of human neuroblastoma SH-SY5Y cells incubated for 24 h with Compound **3h** at different concentrations (range 0.1–50 μM). Untreated cells were used as the control. Results are expressed as the percentage of viable cells observed after treatment with Compound **3h** *vs.* untreated control cells (100%) and shown as the mean ± SD (*n* = 3).

**Figure 2:**
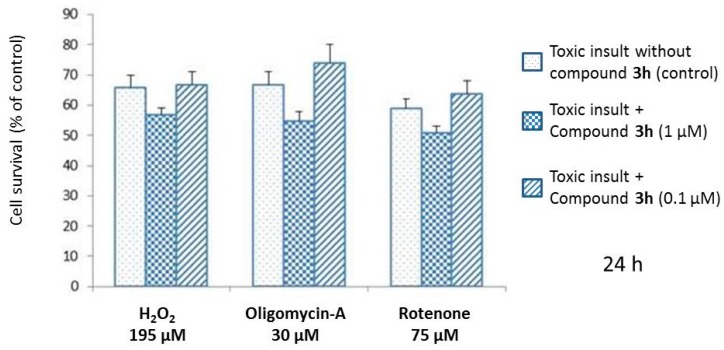
Neuroprotection effect of **3h** on the viability of human neuroblastoma SH-SY5Y cells. Viability was measured after 24 h co-incubation of SH-SY5Y cells with the neurotoxic insult (H_2_O_2_ 195 μM, oligomycin-A 30 μM or rotenone 75 μM) and Compound **3h** at different concentrations (1 µM and 0.1 µM) or in the absence of **3h** (control experiments). Results are expressed as the percentage of viable cells, and data represent the means ± SD (*n* = 3).

On the basis of the enzymatic screening, Compound **3h** came into light as a promising dual AChE-MAO-B inhibitor deserving deeper investigations. Therefore, a preliminary evaluation of the cytotoxicity and neuroprotective efficacy of this hit compound was undertaken in the human SH-SY5Y neuroblastoma cell line by following the 3-(4,5-dimethylthiazol-2-yl)-2,5-diphenyl-tetrazolium bromide (MTT) assay [22]. The viability of SH-SY5Y cells was measured after 24 hours incubation with different concentrations of **3h** ranging from 0.1–50 µM, and untreated cells were used as the control (Figure 1). The compound showed a negligible cytotoxicity also at the highest doses employed in the experiment. In the same SH-SY5Y cell line, the neuroprotective activity of **3h** against three pro-apoptotic insults (hydrogen peroxide, rotenone and oligomycin-A) was studied in separate tests (Figure 2). All of these models of neuronal damage induce oxidative stress conditions and over-production of radicals with different mechanisms. In fact, H_2_O_2_ may act as a radical initiator, producing other harmful reactive oxygen species. On the other hand, both rotenone [23] and oligomycin-A behave as mitochondrial toxins by impairing at different levels the respiratory chain and the ATP production. At a 1 µM concentration, **3h** displayed no increase of cell survival. A moderate neuroprotection against rotenone and oligomycin-A was observed at a lower concentration (0.1 µM).

CNS-active molecules should be able to enter the brain after permeating the blood brain barrier (BBB) by passive diffusion and should be devoid of interactions with glycoprotein-P (P-gp), which limits brain uptake and serves as a defensive efflux mechanism of xenobiotics for the CNS. Brain permeation and P-gp interaction were estimated in a cell-based *in vitro* method using the Madin-Darby canine kidney (MDCK) cell line [24,25]. After transfection with the human MDR1 cDNA (MDCKII-MDR1), this line shows a high expression of P-gp (MDR1) and very tight cellular junctions, thus representing a reliable BBB *in vitro* model. Bidirectional transport for **3h** was evaluated in apical-to-basal (AP) and basal-to-apical (BL) directions, and the measured apparent permeability values (*P*_app_ AP = 2.27 × 10^−5^ cm/s, *P*_app_ BL 2.18 × 10^−5^ cm/s) were superior to those of diazepam (*P*_app_ AP = 1.46 × 10^−5^ cm/s, *P*_app_ BL 1.23 × 10^−5^ cm/s), used as positive CNS-permeant control (Table 2). An efflux ratio (ER) equal to 0.96 and much lower than the threshold value (ER = 2) indicates that **3h** is not likely to be a substrate for P-gp. Taken together, these results pointed out that **3h** might be considered a promising, non-cytotoxic and neuroprotective hit compound endowed with a multi-target profile based on a potent MAO-B inhibition and a moderate AChE affinity. Derivative **3h** can be regarded as a CNS active agent able to rapidly permeate the tight monolayer without interacting with efflux systems and deserves further studies as an anti-Alzheimer agent in animal models.

## 3. Materials and Methods

### 3.1. Chemistry

Starting materials, reagents and analytical-grade solvents were purchased from Sigma-Aldrich (Milan, Italy). The purity of all of the intermediates, checked by ^1^H-NMR and HPLC, was always better than 95%. All of the newly-prepared and tested compounds showed an HPLC purity higher than 98%. Column chromatography was performed using Merck silica gel 60 (0.063–0.200 mm, 70–230 mesh). Flash chromatographic separations were performed on a Biotage SP1 purification system (Biotage Sweden AB, Uppsala, Sweden) using flash cartridges prepacked with KP-Sil 32–63 μm, 60 Å silica. All reactions were routinely checked by TLC using Merck Kieselgel 60 F_254_ aluminum plates and visualized by UV light or iodine. Regarding the reaction requiring the use of dry solvents, the glassware was flame-dried and then cooled under a stream of dry argon before use. Elemental analyses were performed on a EuroEA 3000 analyzer (Eurovector, Milan, Italy) only on the final compounds tested as MAOs and ChEs inhibitors. The measured values for C, H and N agreed to within ±0.40% of the theoretical values. Nuclear magnetic resonance (NMR) spectra were recorded on a Varian Mercury 300 instrument at 300 MHz (Varian, Milan, Italy) at ambient temperature in the specified deuterated solvent. Chemical shifts (δ) are quoted in parts per million (ppm) and are referenced to the residual solvent peak. The coupling constants *J* are given in Hertz (Hz). The following abbreviations were used: s (singlet), d (doublet), dd (doublet of doublet), t (triplet), q (quadruplet), m (multiplet), br s (broad signal); signals due to OH and NH protons were located by deuterium exchange with D_2_O. Melting points were determined by the capillary method on a Stuart Scientific SMP3 electrothermal apparatus (Bibby Scientific, Stone, UK) and are uncorrected.

*7-Methoxy-3-methyl-2H-chromen-2-one* (**1b**): 7-Hydroxy-3-methyl-2*H*-chromen-2-one [15] (3.80 g, 20.0 mmol) was dissolved in anhydrous DMF (70 mL), and potassium carbonate (2.76 g, 20.0 mmol) was added. After stirring for 1 h, methyl iodide (1.49 mL, 24.0 mmol) was dropped via syringe. The mixture was stirred overnight at room temperature and then poured onto crushed ice. After extraction with ethyl acetate (3 × 250 mL), the organic phases were collected and dried over Na_2_SO_4_. Evaporation of the solvent yielded the desired coumarin **1**, which was used without further purification. Yield: 61%. Spectroscopic and analytic data agree with those reported in the literature [26].

*2-Hydroxy-4,5-dimethoxybenzaldehyde*: 2,4,5-Trimethoxybenzaldehyde (3.92 g, 20.0 mmol) was dissolved in dry CH_2_Cl_2_ (20 mL) before adding dropwise boron tribromide (1.0 N in CH_2_Cl_2_, 20 mmol, 20 mL) while cooling at 0 °C. The reaction mixture was slowly warmed at room temperature and left under vigorous magnetic stirring for 20 h. Water (250 mL) was carefully added with simultaneous cooling with an external ice-bath. The suspension was stirred for 1 h and then partitioned with an additional amount of CH_2_Cl_2_ (80 mL). After extraction with CH_2_Cl_2_ (3 × 100 mL), the organic layers were collected and dried over Na_2_SO_4_. The mixture was concentrated to dryness and purified through flash chromatography (gradient eluent: ethyl acetate in *n*-hexane 10%→50%), yielding a yellow solid. Yield: 78%. ^1^H-NMR (DMSO-*d*_6_) δ: 3.71 (3H, s, OCH_3_), 3.80 (3H, s, OCH_3_), 6.53 (1H, s, H-3), 7.11 (s, 1H, H-6), 10.00 (s, 1H, CHO), 10.69 (s, 1H, disappearing with D_2_O, OH).

*6,7-Dimethoxy-3-methyl-2H-chromen-2-one* (**1c**): To a solution of 2-hydroxy-4,5-dimethoxybenzaldehyde (3.29 g, 18.0 mmol) in *N*,*N*-diethylaniline (60 mL), (carbethoxyethylidene) triphenylphosphorane (7.18 g, 19.8 mmol) was added. The mixture was stirred at room temperature for 2 h and then refluxed for 3 h. After cooling at room temperature, HCl (4.0 N in water, 150 mL) was slowly added while cooling at 0 °C, and the resulting suspension was extracted with ethyl acetate (3 × 80 mL). The organic layers were collected, dried over Na_2_SO_4_ and concentrated to dryness under rotary evaporation. The resulting crude oil residue was purified through flash chromatography (gradient eluent: ethyl acetate in *n*-hexane 0%→60%), thus furnishing the desired coumarin **1c** as an off-white solid. Yield: 80%. ^1^H-NMR (DMSO-*d*_6_) δ: 2.04 (3H, s, CH_3_), 3.77 (3H, s, OCH_3_), 3.82 (3H, s, OCH_3_), 7.03 (1H, s, H-8), 7.13 (s, 1H, H-5), 7.74 (s, 1H, H-4).

*3-(Bromomethyl)-2H-chromen-2-one* (**2a**): 3-Methyl-2*H*-chromen-2-one [27] **1a** (1.60 g, 10.0 mmol) was suspended in CCl_4_ (10 mL), and then *N*-bromosuccinimide (1.78 g, 10.0 mmol) and benzoyl peroxide (0.484 g, 1.00 mmol) were added. The mixture was refluxed for 3.5 h, and the succinimide residue was filtered off. The desired bromide crystallized from the hot solution. An additional amount of **2a** was obtained as follows. The mother liquor was concentrated to dryness, thus furnishing a crude solid that was treated with methanol and filtered, yielding **2a** as a yellow solid with satisfactory purity. Yield: 76%. ^1^H-NMR (DMSO-*d*_6_) δ: 4.55 (2H, s, CH_2_Br), 7.37–7.40 (1H, m, H-6), 7.43–7.45 (1H, m, H-8), 7.63–7.66 (1H, m, H-7), 7.74 (1H, dd, *J*_1_ = 7.8 Hz, *J*_2_ = 1.5 Hz, H-5), 8.30 (1H, s, H-4).

*3-(Bromomethyl)-7-methoxy-2H-chromen-2-one* (**2b**): To a stirred suspension of 7-methoxy-3-methyl-2*H*-chromen-2-one (**1b**, 1.82 g, 9.60 mmol) in CCl_4_ (9.6 mL), *N*-bromosuccinimide (2.05 g, 11.5 mmol) and benzoyl peroxide (0.465 g, 1.92 mmol) were added. After heating at reflux for 2 h, the hot reaction mixture was filtered to remove the succinimide by-product, and bromo-derivative **2b** crystallized from CCl_4_ as a yellowish solid. Yield: 62%. ^1^H-NMR (CDCl_3_) δ: 3.89 (3H, s, OCH_3_), 4.43 (2H, s, CH_2_Br), 6.85 (1H, d, *J* = 1.9 Hz, H-8), 6.88 (1H, dd, *J*_1_ = 8.3 Hz, *J*_2_ = 2.5 Hz, H-6), 7.40 (1H, d, *J* = 8.8 Hz, H-5), 7.80 (1H, s, H-4).

*3-(Bromomethyl)-6,7-dimethoxy-2H-chromen-2-one* (**2c**): 6,7-Dimethoxy-3-methyl-2*H*-chromen-2-one (**1c**, 1.98 g, 9.00 mmol) was dissolved in hot CCl_4_ (9 mL). *N*-Bromosuccinimide (1.69 g, 9.50 mmol) was added followed by benzoyl peroxide (0.218 g, 0.90 mmol), while heating. The reaction mixture was refluxed for 7 h. After cooling at room temperature, the solvent was removed under reduced pressure. The resulting crude solid was treated with methanol, filtered and washed with hot ethanol, yielding **2c** as a yellow solid. Yield: 77%. ^1^H-NMR (CDCl_3_) δ: 3.92 (3H, s, OCH_3_), 3.96 (3H, s, OCH_3_), 4.44 (2H, s, CH_2_Br), 6.85 (1H, s, H-8), 6.86 (1H, s, H-5), 7.78 (1H, s, H-4).

General procedure for the synthesis of amines **3a**, **3l** and **3m**: GlyNH_2_∙HCl (for **3a**, **3l**, 0.464 g, 4.20 mmol) or GlyNHCH_3_∙HCl (for **3m**, 0.523 g, 4.20 mmol) were suspended in DMF (4 mL) with triethylamine (0.585 mL, 4.20 mmol) under vigorous magnetic stirring while heating at 70 °C. The suitable bromide **2a** (0.143 g, 0.60 mmol) or **2c** (0.179 g, 0.60 mmol), previously dissolved in DMF (2 mL), was added portion-wise within 3.5 h (0.250 mL every 30 min). The mixture was stirred at 70 °C for an additional 4 h. After cooling at room temperature, brine (50 mL) was added, and the mixture was extracted with ethyl acetate (3 × 30 mL). The organic layers were collected, washed with brine (3 × 20 mL), dried over Na_2_SO_4_ and concentrated under reduced pressure. The crude mixtures were further purified as indicated below.

*N^2^-[(2-oxo-2H-Chromen-3-yl)methyl]glycinamide* (**3a**): Purification through flash chromatography (gradient eluent: methanol in chloroform 0%→5%). Yield: 62%. Mp: 149 (dec.), 159–160 °C. ^1^H-NMR (DMSO-*d*_6_) δ: 2.62 (1H, s, NH; dis. with D_2_O), 3.11 (2H, s, CH_2_CO), 3.54 (2H, s, C*H*_2_NH), 7.07 (1H, s, NH; dis. with D_2_O), 7.33–7.36 (2H, m, NH, H-6; 1H dis. with D_2_O), 7.40 (1H, d, *J* = 8.3 Hz, H-8), 7.57 (1H, t, *J* = 7.8 Hz, H-7), 7.70 (1H, d, *J* = 7.3 Hz, H-5), 8.00 (1H, s, H-4). Anal. C 62.41, H 5.26, N 12.00%, calcd. for C_12_H_12_N_2_O_3_, C 62.06, H 5.21, N 12.06%.

*N^2^-[(6,7-Dimethoxy-2-oxo-2H-chromen-3-yl)methyl]glycinamide* (**3l**): Purification through flash chromatography (gradient eluent: methanol in chloroform 0%→15%) afforded a solid that was further crystallized from hot ethanol 95°. Yield: 57%. Mp: 176–178 °C. ^1^H-NMR (DMSO-*d*_6_) δ: 3.09 (2H, s, CH_2_CO), 3.49 (2H, s, C*H*_2_NH), 3.79 (3H, s, OCH_3_), 3.84 (3H, s, OCH_3_), 7.05 (1H, s, H-8), 7.06 (1H, s, NH; dis. with D_2_O), 7.23 (1H, s, H-5), 7.34 (1H, s, NH; dis. with D_2_O), 7.90 (1H, s, H-4); NH not detected. Anal. C 57.86, H 5.27, N 9.24%, calcd. for C_14_H_16_N_2_O_5_, C 57.53, H 5.52, N 9.58%.

*N^2^-[(6,7-Dimethoxy-2-oxo-2H-chromen-3-yl)methyl]-N^1^-methylglycinamide* (**3m**): Purification through flash chromatography (gradient eluent: methanol in chloroform 0%→10%) afforded a solid that was further crystallized from hot ethanol. Yield: 65%. Mp: 147–149 °C. ^1^H-NMR (DMSO-*d*_6_) δ: 2.59 (3H, d, *J* = 4.7 Hz, C*H*_3_NH), 3.10 (2H, s, CH_2_CO), 3.48 (2H, s, C*H*_2_NH), 3.79 (3H, s, OCH_3_), 3.84 (3H, s, OCH_3_), 7.06 (1H, s, H-8), 7.21 (1H, s, H-5), 7.82 (1H, br q, *J* = 4.7 Hz, CH_3_N*H*; dis. with D_2_O), 7.89 (1H, s, H-4); NH not detected. Anal. C 59.07, H 5.65, N 8.93%, calcd. for C_15_H_18_N_2_O_5_, C 58.82, H 5.92, N 9.15%.

General procedure for the synthesis of amines **3b**, **3d**, **3e** and **3g**: Triethylamine (0.335 mL, 2.40 mmol) and suitable substituted *N*-benzylmethylamine hydrochlorides [15] (0.72 mmol) were added to a solution of the appropriate bromo-intermediate **2a**–**c** (0.60 mmol) in THF (5 mL). After stirring at room temperature for 24 h, the inorganic residue was filtered-off, and the solvent was evaporated under vacuum. Flash chromatography purification afforded the desired compounds with different eluting conditions illustrated as follows.

*3-{[(3-Fluorobenzyl)(methyl)amino]methyl}-2H-chromen-2-one hydrochloride* (**3b**): Purification through flash chromatography (gradient eluent: ethyl acetate in *n*-hexane 0%→70%) afforded an oil that was transformed into the corresponding hydrochloride by treatment with the commercially available solution of HCl 4.0 N in dioxane. Yield: 60%. Mp: 236–237 °C. ^1^H-NMR (DMSO-*d*_6_) δ: 2.71 (3H, s, NCH_3_), 4.16–4.26 (2H, m, CH_2_), 4.36–4.39 (1H, m, Ar-CHa), 4.51–4.54 (1H, m, Ar-CHb), 7.30–7.33 (1H, m, H-6), 7.37–7.53 (5H, m, C_6_H_4_, H-8), 7.71 (1H, t, *J* = 7.3 Hz, H-7), 7.77 (1H, d, *J* = 7.3 Hz, H-5), 8.33 (1H, s, H-4), 10.02 (1H, s, NH^+^; dis. with D_2_O). Anal. C 65.02, H 4.96, N 4.00%, calcd. for C_18_H_17_FClNO_2_, C 64.77, H 5.13, N 4.20%.

*3-{[(3-Fluorobenzyl)(methyl)amino]methyl}-7-methoxy-2H-chromen-2-one* (**3d**): Purification through flash chromatography (gradient eluent: ethyl acetate in dichloromethane 0%→20%). Yield: 74%. Oil compound. ^1^H-NMR (DMSO-*d*_6_) δ: 2.17 (3H, s, NCH_3_), 3.38 (2H, s, CH_2_), 3.61 (2H, s, Ar-CH_2_), 3.84 (3H, s, OCH_3_), 6.94 (1H, dd, *J*_1_ = 8.8 Hz, *J*_2_ = 2.5 Hz, H-6), 6.98 (1H, d, *J* = 1.9 Hz, H-8), 7.03–7.07 (1H, m, Ar-CHa), 7.19–7.20 (2H, m, Ar-CHb, Ar-CHc), 7.33–7.37 (1H, m, Ar-CHd), 7.68 (1H, d, *J* = 8.8 Hz, H-5), 7.98 (1H, s, H-4). Anal. C 70.03, H 5.59, N 4.39%, calcd. for C_19_H_18_FNO_3_, C 69.71, H 5.54, N 4.28%.

*3-{[[(7-Methoxy-2-oxo-2H-chromen-3-yl)methyl](methyl)amino]methyl}benzonitrile* (**3e**): Purification through flash chromatography (gradient eluent: ethyl acetate in dichloromethane 0%→20%) afforded an oil that was treated with diethyl ether and furnished the desired product as a yellow solid. Yield: 88%. Mp: 123–125 °C. ^1^H-NMR (DMSO-*d*_6_) δ: 2.16 (3H, s, NCH_3_), 3.39 (2H, s, CH_2_), 3.65 (2H, s, Ar-CH_2_), 3.84 (3H, s, OCH_3_), 6.94 (1H, dd, *J*_1_ = 8.8 Hz, *J*_2_ = 2.4 Hz, H-6), 6.98 (1H, d, *J* = 2.4 Hz, H-8), 7.53 (1H, t, *J* = 7.3 Hz, H-5a), 7.67 (1H, d, *J* = 8.8 Hz, H-5), 7.70–7.72 (2H, m, H-4a, H-6a), 7.81 (1H, s, H-2a), 7.98 (1H, s, H-4). Anal. C 72.09, H 5.38, N 8.15%, calcd. for C_20_H_18_N_2_O_3_, C 71.84, H 5.43, N 8.38%.

*3-{[(3-Chlorobenzyl)(methyl)amino]methyl}-6,7-dimethoxy-2H-chromen-2-one hydrochloride* (**3g**): Purification through flash chromatography (gradient eluent: ethyl acetate in *n*-hexane 0%→60%) afforded an oil that was dissolved in dioxane and transformed into the corresponding hydrochloride by treatment with the commercially available solution of HCl 4.0 N in dioxane. Yield: 96%. Mp: 229–230 °C. ^1^H-NMR (DMSO-*d*_6_) δ: 2.65 (3H, br s, NCH_3_), 3.81 (3H, s, OCH_3_), 3.87 (3H, s, OCH_3_), 4.14–4.18 (2H, m, CH_2_), 4.29–4.35 (1H, m, Ar-CHaN), 4.47–4.51 (1H, m, Ar-CHbN), 7.15 (1H, s, H-8), 7.23 (1H, s, H-2a), 7.45–7.58 (3H, m, H-4a, H-5a, H-6a), 7.73 (1H, s, H-5), 8.24 (1H, s, H-4), 10.35 (1H, br s, NH^+^; dis. with D_2_O). Anal. C 58.09, H 5.08, N 3.27%, calcd. for C_20_H_21_Cl_2_NO_4_, C 58.55, H 5.16, N 3.41%.

General procedure for the synthesis of amines **3c**, **3f** and **3h**: Appropriate bromide **2a**–**c** (0.6 mmol) was dissolved in THF (3 mL), and K_2_CO_3_ (0.083 g, 0.6 mmol) and *N*-methylpropargylamine (0.10 mL, 1.2 mmol) were added. After stirring for 6 h at room temperature, the inorganic residue was filtered off, and the resulting mixture was purified as described below.

*3-{[Methyl(prop-2-yn-1-yl)amino]methyl}-2H-chromen-2-one* (**3c**): Purified through crystallization from ethanol/water. Yield: 46%. Mp: 77–78 °C. ^1^H-NMR (acetone-*d*_6_) δ: 2.38 (3H, s, NCH_3_), 2.75 (1H, t, *J* = 2.3 Hz, C≡CH), 3.45 (2H, d, *J* = 2.3 Hz, C*H*_2_-C≡CH), 3.50 (2H, s, C*H*_2_-N(CH_3_)-CH_2_-C≡CH), 7.33–7.36 (2H, m, H-6, H-8), 7.55–7.61 (1H, m, H-7), 7.70 (1H, dd, *J*_1_ = 8.2 Hz, *J*_2_ = 1.8 Hz, H-5), 7.92 (1H, s, H-4). Anal. C 74.36, H 5.55, N 6.01%, calcd. for C_14_H_13_NO_2_, C 73.99, H 5.77, N 6.16%.

*7-Methoxy-3-{[methyl(prop-2-yn-1-yl)amino]methyl}-2H-chromen-2-one hydrochloride* (**3f**): Purification through column chromatography (gradient eluent: ethyl acetate in *n*-hexane 40%→70%) afforded an oil that was transformed into the corresponding hydrochloride as follows with the commercially available solution of HCl 4.0 N in dioxane. Yield: 66%. Mp: 203–205 °C (dec.). ^1^H-NMR (DMSO-*d*_6_) δ: 2.76 (3H, s, NCH_3_), 3.86 (4H, br s, C≡CH, OCH_3_), 4.11–4.14 (4H, m, C*H*_2_-NH^+^-C*H*_2_), 6.99 (1H, dd, *J*_1_ = 8.8 Hz, *J*_2_ = 2.3 Hz, H-6), 7.07 (1H, d, *J* = 2.3 Hz, H-8), 7.68 (1H, d, *J* = 8.2 Hz, H-5), 8.22 (1H, s, H-4), 10.50 (1H, br s, NH^+^; dis. with D_2_O). Anal. C 61.49, H 5.49, N 4.49%, calcd. for C_15_H_16_ClNO_3_, C 61.33, H 5.49, N 4.77%.

*6,7-Dimethoxy-3-{[methyl(prop-2-yn-1-yl)amino]methyl}-2H-chromen-2-one hydrochloride* (**3h**): Purification through flash chromatography (gradient eluent: ethyl acetate in *n*-hexane 20%→70%) afforded a yellow solid that was transformed into the corresponding hydrochloride with the commercially available solution of HCl 4.0 N in dioxane. Yield: 92%. Mp: 223–225 °C. ^1^H-NMR (DMSO-*d*_6_) δ: 2.76 (3H, s, NCH_3_), 3.81 (3H, s, OCH_3_), 3.87 (3H, s, OCH_3_), 4.11 (4H, br s, C*H*_2_-NH^+^-C*H*_2_), 7.15 (1H, s, H-8), 7.25 (1H, s, H-5), 8.19 (1H, s, H-4), 10.52 (1H, s, NH^+^; dis. with D_2_O); C≡CH not detected. Anal. C 59.58, H 5.41, N 4.26%, calcd. for C_16_H_18_ClNO_4_, C 59.35, H 5.60, N 4.33%.

General procedure for the synthesis of amines **3i**, **3j** and **3k**: The appropriate amine (for **3i**: commercially available methylamine 2.0 N in THF, 1.5 mL, 3.0 mmol; for **3j**: benzylamine, 0.328 mL, 3.0 mmol; for **3k**: 4-(aminomethyl)benzonitrile hydrochloride [15], 0.506 g, 3.0 mmol) was diluted or suspended in THF (2.2 mL), and K_2_CO_3_ (0.138 g, 1.0 mmol) was added. The mixture was kept at room temperature under magnetic stirring, and bromide **2c** (0.155 g, 0.50 mmol), previously dissolved in THF (2.8 mL), was added in small aliquots every 30 min (7 portions, 0.40 mL each). The mixture was stirred for an additional 30 min. The inorganic residue was filtered, and the resulting solution was concentrated under vacuum and purified through flash chromatography as detailed below.

*6,7-Dimethoxy-3-[(methylamino)methyl]-2H-chromen-2-one hydrochloride* (**3i**): Purification through flash chromatography (gradient eluent: methanol in ethyl acetate 0%→5%) afforded an oil that was transformed into the corresponding hydrochloride with the commercially available solution of HCl 1.25 N in ethanol. Yield: 92%. Mp: 215–217 °C. ^1^H-NMR (DMSO-*d*_6_) δ: 2.59 (3H, s, NCH_3_), 3.81 (3H, s, OCH_3_), 3.87 (3H, s, OCH_3_), 3.99 (2H, s, NCH_2_), 7.15 (1H, s, H-8), 7.25 (1H, s, H-5), 8.11 (1H, s, H-4), 8.76 (2H, br s, NH_2_^+^; dis. with D_2_O). Anal. C 54.90, H 5.36, N 4.77%, calcd. for C_13_H_16_ClNO_4_, C 54.65, H 5.64, N 4.90%.

*3-[(Benzylamino)methyl]-6,7-dimethoxy-2H-chromen-2-one* (**3j**): Purification through flash chromatography (gradient eluent: methanol in ethyl acetate 0%→5%). Yield: 88%. Mp: 112–114 °C. ^1^H-NMR (DMSO-*d*_6_): δ 2.62 (1H, br s, NH), 3.49 (2H, s, NCH_2_), 3.74 (2H, s, Ar-CH_2_), 3.79 (3H, s, OCH_3_), 3.83 (3H, s, OCH_3_), 7.05 (1H, s, H-8), 7.27 (1H, s, H-5), 7.19–7.38 (5H, m, Ph), 7.92 (1H, s, H-4). Anal. C 70.23, H 5.72, N 4.17%, calcd. for C_19_H_19_NO_4_, C 70.14, H 5.89, N 4.31%.

*4-({[(6,7-Dimethoxy-2-oxo-2H-chromen-3-yl)methyl]amino}methyl)benzonitrile* (**3k**): Purification through flash chromatography (gradient eluent: methanol in ethyl acetate 0%→5%). Yield: 93%. Mp: 146–148 °C. ^1^H-NMR (DMSO-*d*_6_) δ: 3.49 (2H, s, NCH_2_), 3.79 (3H, s, OCH_3_), 3.83–3.85 (5H, m, Ar-CH_2_, OCH_3_), 7.06 (1H, s, H-8), 7.25 (1H, s, H-5), 7.58 (2H, d, *J* = 8.3 Hz, H-3a, H-5a), 7.78 (2H, d, *J* = 8.3 Hz, H-2a, H-6a), 7.90 (1H, s, H-4); NH not detected. Anal. C 68.88, H 5.06, N 7.94%, calcd. for C_20_H_18_N_2_O_4_, C 68.56, H 5.18, N 8.00%.

*2,4-Dihydroxy-5-methoxybenzaldehyde* (**4**) [18]: In a flame-dried round-bottom flask, dry AlCl_3_ (8.00 g, 60.0 mmol) was suspended in dry dichloromethane (30 mL), and a solution of 2,4,5-trimethoxybenzaldehyde (1.96 g, 10.0 mmol) in dry dichloromethane (20 mL) was added dropwise while cooling at 0 °C with an external ice bath. The mixture was then refluxed for 4 h and poured onto crushed ice (100 g). Concentrated HCl (10 mL) was carefully added, and the resulting suspension was vigorously stirred for additional 15 min. After extraction with dichloromethane (3 × 50 mL), the organic layers were collected and dried over Na_2_SO_4_. The solvent was removed under rotary evaporation, and the resulting crude was crystallized from hot toluene. Yield: 75%. Spectroscopic and analytic data agree with those reported in the literature [18].

*7-Hydroxy-6-methoxy-3-methyl-2H-chromen-2-one* (**5**): Aldehyde **4** (0.841 g, 5.00 mmol) was dissolved in dry *o*-xylene (25 mL) before the addition of (carbethoxyethylidene)triphenylphosphorane (2.72 g, 7.50 mmol). After heating at reflux for 12 h, the solvent was evaporated under vacuum. Column chromatography purification (eluent: ethyl acetate in *n*-hexane 40%, *v*/*v*) afforded the desired coumarin. Yield: 56%. ^1^H-NMR (acetone-*d*_6_) δ: 2.09 (3H, s, CH_3_), 3.90 (3H, s, OCH_3_), 6.79 (1H, s, H-8), 7.12 (1H, s, H-5), 7.65 (1H, s, H-4); OH not detected.

*6-Methoxy-3-methyl-2-oxo-2H-chromen-7-yl benzoate* (**6**): Intermediate **5** (0.516 g, 2.50 mmol), was dissolved in dry THF (20 mL), and triethylamine (0.522 mL, 3.75 mmol) was added. The solution was cooled at 0 °C, and benzoyl chloride (0.435 mL, 3.75 mmol) was added dropwise. After 10 min, the cooling bath was removed, and the mixture was stirred at room temperature for 5 h. The resulting suspension was diluted with dichloromethane (100 mL) and washed with saturated aqueous NaHCO_3_ (3 × 40 mL). The organic phase was dried over Na_2_SO_4_ and filtered, and the solvent was evaporated under vacuum. The residue purified through flash chromatography (gradient eluent: ethyl acetate in *n*-hexane 0%→40%). Yield: 59%. ^1^H-NMR (CDCl_3_) δ: 2.84 (3H, s, CH_3_), 3.87 (3H, s, OCH_3_), 7.33 (1H, s, H-8), 7.61 (2H, t, *J* = 7.8 Hz, H-3a, H-5a), 7.73–7.77 (2H, m, H-5, H-4a), 7.96 (1H, s, H-4), 8.18 (2H, d, *J* = 7.3 Hz, H-2a, H-6a).

*3-(Bromomethyl)-6-methoxy-2-oxo-2H-chromen-7-yl benzoate* (**7**): *N*-Bromosuccinimide (0.214 g, 1.20 mmol) was added to a suspension of 6-methoxy-3-methyl-2-oxo-2*H*-chromen-7-yl benzoate **6** (0.310 g, 1.00 mmol) in CCl_4_ (2 mL) followed by benzoyl peroxide (0.073 g, 0.30 mmol). The mixture was refluxed for 12 h under magnetic stirring. After the removal of the solvent under rotary evaporation, the solid residue was purified through flash chromatography (gradient eluent: CH_2_Cl_2_ in *n*-hexane 40%→90%). Yield: 39%. ^1^H-NMR (CDCl_3_) δ: 3.88 (3H, s, OCH_3_), 4.45 (2H, s, BrCH_2_), 7.04 (1H, s, H-8), 7.24 (1H, s, H-5), 7.55 (2H, t, *J* = 7.3 Hz, H-3a, H-5a), 7.66–7.69 (1H, m, H-4a), 7.86 (1H, s, H-4), 8.21–8.23 (2H, m, H-2a, H-6a).

*7-Hydroxy-6-methoxy-3-{[methyl(prop-2-yn-1-yl)amino]methyl}-2H-chromen-2-one* (**8**): Bromide **7** (0.117 g, 0.30 mmol) was dissolved in THF (2 mL) before adding K_2_CO_3_ (0.415 g, 3.0 mmol) and *N*-methylpropargylamine (0.051 mL, 0.60 mmol). The mixture was stirred at room temperature overnight, and the inorganic residue was filtered off after washing with THF. The resulting solution was concentrated under rotary evaporation and purified through flash chromatography (gradient eluent: ethyl acetate in *n*-hexane 10%→60%). Yield: 91%. Mp: 161 (dec.), 167–168 °C. ^1^H-NMR (DMSO-*d*_6_) δ: 2.25 (3H, s, NCH_3_), 3.18 (1H, t, *J* = 2.4 Hz, C≡CH), 3.35 (2H, s, C*H*_2_-N(CH_3_)-CH_2_-C≡CH), 3.36 (2H, d, *J* = 2.4 Hz, C*H*_2_-C≡CH), 3.79 (3H, s, OCH_3_), 6.75 (1H, s, H-8), 7.25 (1H, s, H-5), 7.80 (1H, s, H-4), 10.20 (1H, s, OH; dis. with D_2_O). Anal. C 66.14, H 5.33, N 5.07%, calcd. for C_15_H_15_NO_4_, C 65.92, H 5.53, N 5.13%.

### 3.2. Monoamine Oxidase Inhibition Assays

The *r*MAO inhibitory activity of compounds in Table 1 was assessed using a continuous spectrophotometric assay [28], monitoring the rate of oxidation of the nonselective nonfluorescent MAO substrate kynuramine to 4-hydroxyquinoline, as previously reported [21]. Propargylamine-bearing inhibitors **3c**, **3f**, **3h** and **8** were pre-incubated with the enzyme preparation for 30 min before the addition of kynuramine and determination of MAO activity. Finally, IC_50_ values were determined by nonlinear regression of MAO inhibition *vs.* -log of the concentration plots, using the program Origin, Version 6.0 (Microcal Software Inc., Northampton, MA, USA).

### 3.3. Cholinesterases Inhibition Assays

The spectrophotometric Ellman’s test [20] for the *in vitro* inhibition assay of AChE from electric eel (463 U/mg; Sigma Aldrich, Milan, Italy) and BChE from equine serum (13 U/mg; Sigma) was followed as previously described [29]. The concentration of compound that determined 50% inhibition of the ChE activity (IC_50_) was calculated by non-linear regression of the response-log(concentration) curve, using GraphPad Prism v. 5.

### 3.4. Cytotoxicity Assays

Human neuroblastoma cells SH-SY5Y were maintained at 37 °C in a humidified incubator containing 5% CO_2_ in DMEM nutrient (Lonza) supplemented with 10% heat inactivated FBS, 2 mM L-glutamine, 100 U/mL penicillin and 100 µg/mL streptomycin. Cells were dispensed into 96-well microtiter plates at a density of 10,000 cells/well. Following overnight incubation, cells were treated with a range of different concentrations of Compound **3h** (0.1–50 µM). Then, the plates were incubated at 37 °C for 24 h. An amount of 10 µL of 0.5% *w*/*v* MTT was further added to each well, and the plates were incubated for an additional 3 h at 37 °C. Finally, the cells were lysed by the addition of 100 µL of DMSO/EtOH 1:1 (*v*/*v*) solution. The absorbance at 570 nm was determined using a Perkin Elmer 2030 multi-label reader Victor TM X3 (Perkin Elmer, Milan, Italy).

### 3.5. Neuroprotection against Oxidative Stress Insults

Human neuroblastoma SH-SY5Y cells were dispensed into 96-well microtiter plates at a density of 10,000 cells/well. After 24 h of incubation at 37 °C in a humidified incubator containing 5% CO_2_, cells were co-incubated with H_2_O_2_ (195 µM) or oligomycin A (30 µM) or rotenone (75 µM), and compounds were tested at several concentrations (0.1 µM and 1 µM) for further 24 h. The cell viability was determined by the MTT assay and analyzed as above described. Each compound was tested in triplicate, and the experiments were repeated three times. Statistical significance was assigned to *p* < 0.01 and calculated using a one-way analysis of variance (ANOVA) followed by the Bonferroni *post hoc* tests (GraphPad Prism vers. 5). Where indicated, the standard error of the mean (SD) for data points has been calculated, and the number of experiments is given (n).

### 3.6. Bidirectional Transport Studies

MDCKII-MDR1 cells were cultured in DMEM medium and seeded at a density of 100,000 cell/cm^2^ onto polyester 12 well Transwell inserts (pore size: 0.4 µm; 12 mm diameter; apical volume: 0.5 mL; basolateral: volume 1.5 mL). MDCKII-MDR1 cell barrier function was verified prior to the described transport experiments by means of trans-epithelial electrical resistance (TEER) using an EVOM apparatus and the measurement of the flux of fluorescein isothiocyanate-dextran (FD4, Sigma Aldrich, Milan, Italy) (200 µg/mL) and diazepam (75 µM). The TEER was measured in growth media (DMEM) at room temperature and calculated as the measured resistance minus the resistance of an empty Transwell (blank without cells). Cell monolayers with TEER values 800 Ohm/cm^2^ were used. Following the TEER measurements, the cells were equilibrated in transport medium in both the apical and basolateral chambers for 30 min at 37 °C. The composition of transport medium was as follows: 0.4 mM K_2_HPO_4_, 25 mM NaHCO_3_, 3 mM KCl, 122 mM NaCl, 10 mM glucose, pH = 7.4. The osmolarity was equal to 300 mOsm, as determined by a freeze point-based osmometer. At Time 0, culture medium was aspirated from both the AP and BL chambers of each insert, and cell monolayers were washed three times (10 min per wash) with Dulbecco’s Phosphate-Buffered Saline (DPBS) pH = 7.4. Finally, a solution of compound diluted in transport medium was added to the apical or basolateral chamber. For AP-to-BL or BL-to-AP flux studies, the drug solution was added in the AP chamber or in the BL chamber, respectively. Except for FD4, which was solubilized directly in the assay medium at a concentration of 200 µg/mL, the other compounds were first dissolved in DMSO and then diluted with the assay medium to a final concentration of 75 µM. Next, the tested solutions were added to the donor side (0.5 mL for the AP chamber and 1.5 mL for the BL chamber), and fresh assay medium was placed in the receiver compartment. The percentage of DMSO never exceeded 1% (*v*/*v*) in the samples. The transport experiments were carried out under cell culture conditions (37 °C, 5% CO_2_, 95% humidity). After an incubation time of 120 min, samples were removed from the apical and basolateral side of the monolayer and then stored until further analysis.

Quantitative analysis of Compounds **3h** and diazepam was performed through UV-visible (VIS) spectroscopy using a PerkinElmer double-beam UV-visible spectrophotometer Lambda Bio 20 (Perkin Elmer, Milan, Italy), equipped with 10-mm path-length-matched quartz cells. Standard calibration curves were prepared at the maximum absorption wavelength of each compound using PBS as the solvent and were linear (*r*^2^ = 0.999) over the range of tested concentrations (from 5–100 µM). The FD4 samples were analyzed with a Victor3 fluorimeter (Wallac Victor3, 1420 Multilabel Counter, Perkin Elmer) at excitation and emission wavelengths of 485 and 535 nm, respectively. Each compound was tested in triplicate, and the experiments were repeated three times.

The apparent permeability, in units of cm/s, was calculated using the following equation:

$$P_{app}=(\frac{V_{A}}{area\times time})\times (\frac{{\left[drug\right]}_{acceptor}}{{\left[drug\right]}_{initial}})$$

where “VA” is the volume in the acceptor well, “area” is the surface area of the membrane, “time” is the total transport time, “[drug]_acceptor_” is the concentration of the drug measured by UV-spectroscopy and “[drug]_initial_” is the initial drug concentration in the AP or BL chamber. The efflux ratio (ER) was calculated using the following equation: ER = *P*_app_, BL-AP/*P*_app_, AP-BL, where *P*_app_, BL-AP is the apparent permeability of basal-to-apical transport and *P*_app_, AP-BL is the apparent permeability of apical-to-basal transport. An efflux ratio greater than 2 indicates that a test compound is likely to be a substrate for P-gp transport.

## 4. Conclusions

The discovery of novel therapeutics able to halt or reverse the neurotoxic cascade in Alzheimer’s disease still represents an unmet goal. Starting from our expertise in the field of the chemical decoration of the coumarin backbone and in the structure-based design of MAO and AChE inhibitors, herein, we described the development of novel multi-target ligands able to potentially improve the cognitive and learning symptoms of AD (by inhibiting AChE), to reduce the production of ROS and ameliorate oxidative stress neuronal conditions through the inhibition of MAO-B activity, taking advantage of a synergy of actions against the disease. Limited structural variations were studied at positions 6 and 7 of the coumarin backbone, where the presence of two methoxy groups strongly improved MAO-B affinity. The molecular framework was essentially based on the presence of a variously-substituted basic head at position 3 with different steric, stereoelectronic and hydrogen bonding features. The *in vitro* biological screening towards ChEs and MAOs led to the identification of a hit compound (**3h**) endowed with a good AChE affinity and acting as a putative “suicide-type” MAO-B inhibitor with an outstanding potency in the low nanomolar range. Such irreversible mechanism of inhibition, deriving from the formation of a covalent bond between the flavin cofactor and the alkyne group, has widely been documented in the literature for propargylamine inhibitors of MAO [30,31]. In addition, the extraordinarily high MAO-B/-A selectivity promises a safe *in vivo* profile devoid of cheese-effect toxicity. The interesting multi-target profile of **3h** prompted us to study its intrinsic cytotoxicity and to analyze its behavior in the presence of different oxidative insults. In cell-based assays, **3h** displayed a wide therapeutic index and moderately protected neurons against toxicity induced by two respiratory chain breakers, *i.e.*, oligomycin-A and rotenone. Bidirectional transport studies proved its ability to rapidly permeate BBB without suffering interactions with the P-gp-mediated efflux system, thus endorsing its potential application in the treatment of neurodegenerative disorders.

## Figures and Tables

**Table 1:** Monoamine oxidase (MAO) and cholinesterase (AChE, BChE) inhibition data of Compounds **3a**–**m** and **8**. 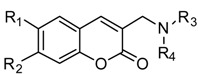

Comp.	R_1_	R_2_	R_3_	R_4_	IC_50_ (µM) or % Inhibition at 10 µM ^a^			
					MAO-A ^b^	MAO-B ^b^	AChE ^c^	BChE ^d^
**3a**	H	H	H	CH_2_CONH_2_	<5%	<5%	12 ± 1	6.2% ± 0.3%
**3b** ^e^	H	H	Me	3′-FBn	<5%	<5%	6.8 ± 0.1	15% ± 2%
**3c**	H	H	Me	CH_2_C≡CH	7.0 ± 1.4 ^f^	0.377 ± 0.014 ^f^	8.4 ± 0.5	<5%
**3d**	H	OMe	Me	3′-FBn	6.0% ± 0.8%	<5%	6.6 ± 0.1	8.5% ± 2.4%
**3e**	H	OMe	Me	3′-CNBn	9.2% ± 1.0%	<5%	8.4 ± 0.3	<5%
**3f** ^e^	H	OMe	Me	CH_2_C≡CH	0.669% ± 0.010 ^f^	0.214 ± 0.008 ^f^	14 ± 1	13% ± 1%
**3g** ^e^	OMe	OMe	Me	3′ClBn	7.0% ± 1.1%	<5%	4.4 ± 1.1	19% ± 2%
**3h** ^e^	OMe	OMe	Me	CH_2_C≡CH	<5% ^f^	0.0028 ± 0.0004 ^f^	9.0 ± 0.5	<5%
**3i** ^e^	OMe	OMe	H	Me	<5%	<5%	13 ± 1	10% ± 2%
**3j**	OMe	OMe	H	Bn	<5%	<5%	2.8 ± 0.2	27% ± 1%
**3k**	OMe	OMe	H	4′CNBn	20% ± 3%	<5%	8.4 ± 1.1	13% ± 2%
**3l**	OMe	OMe	H	CH_2_CONH_2_	<5%	<5%	8.0 ± 1.2	10% ± 1%
**3m**	OMe	OMe	H	CH_2_CONHMe	<5%	<5%	7.7 ± 0.4	11% ± 1%
**8**	OMe	OH	Me	CH_2_C≡CH	35% ± 2% ^f^	11% ± 1% ^f^	9.3 ± 0.4	<5%
Donepezil					-	-	0.020 ± 0.002	2.3 ± 0.1
Rasagiline					0.412 + 0.123 ^g^	0.0044 + 0.0009 ^g^	-	-

^a^ Values are mean of three independent experiments; ^b^ from rat brain homogenates; ^c^ from electric eel; ^d^ from equine serum; ^e^ tested as hydrochloride salt; ^f^ determined after 30 min pre-incubation; ^g^ data taken from [19].

**Table 2:** Bi-directional transport of **3h** and control compounds across Madin-Darby canine kidney (MDCK)II-MDR1 cells.

Compd.	*P_app_* AP (cm/s)	*P_app_* BL (cm/s)	ER ^a^ *P*_app_ BL/*P*_app_ AP
**3h**	2.27 × 10^−5^	2.18 × 10^−5^	0.96
diazepam	1.46 × 10^−5^	1.23 × 10^−5^	0.84
FD-4	1.03 × 10^−6^	2.08 × 10^−7^	0.20

^a^ Efflux ratio (ER) was calculated using the following equation: ER = *P*_app_ BL/*P*_app_ AP, where *P*_app_ BL is the apparent permeability of basal-to-apical transport and *P*_app_ AP is the apparent permeability of apical-to-basal transport. An efflux ratio greater than 2 indicates that a test compound is likely to be a substrate for P-gp transport. Fluorescein isothiocyanate-dextran (FD4) and diazepam were used as paracellular and transcellular markers, respectively, of cell monolayers’ integrity and as an internal control to verify tight junction integrity during the assay.
